# Loss of the mitochondrial kinase PINK1 does not alter platelet function

**DOI:** 10.1038/s41598-018-32716-4

**Published:** 2018-09-26

**Authors:** Tony G. Walsh, Marion T. J. van den Bosch, Kirsty E. Lewis, Christopher M. Williams, Alastair W. Poole

**Affiliations:** 1 School of Physiology, Pharmacology and Neuroscience, Biomedical Sciences Building, University of Bristol, Bristol, BS8 1TD United Kingdom; 2 Present Address: InteRNA Technologies BV, Utrecht, 3584 CM The Netherlands

## Abstract

PTEN-induced putative kinase (PINK) 1 is regarded as a master regulator of cellular mitophagy such that loss of function mutations contribute to early onset Parkinson’s disease, through aberrant mitochondrial control and function. Mitochondrial function is key to platelet procoagulant activity, controlling the haemostatic response to vessel injury, but can also predispose blood vessels to thrombotic complications. Here, we sought to determine the role of PINK1 in platelet mitochondrial health and function using PINK1 knockout (KO) mice. The data largely show an absence of such a role. Haematological analysis of blood counts from KO mice was comparable to wild type. Quantification of mitochondrial mass by citrate synthase activity assay or expression of mitochondrial markers were comparable, suggesting normal mitophagy in KO platelets. Analysis of mitochondrial permeability transition pore opening, changes in mitochondrial membrane potential and calcium signalling to platelet activation were unaffected by loss of PINK1, whereas subtle enhancements of activation-induced reactive oxygen species were detected. Platelet aggregation, integrin activation, α- and dense granule secretion and phosphatidylserine exposure were unaltered in KO platelets while mouse tail bleeding responses were similar to wild type. Together these results demonstrate that PINK1 does not regulate basal platelet mitophagy and is dispensable for platelet function.

## Introduction

PTEN-induced putative kinase 1 (PINK1) is a 63 kDa serine/threonine kinase known for its protective role in eukaryotic cells by regulating the selective removal of damaged mitochondria (mitophagy)^1^. It is well accepted that aberrant mitochondrial function and dynamics are the hallmarks of numerous neurodegenerative disorders and more specifically, loss of function mutations in the catalytic C-terminus domain of PINK1, have been causally associated with early onset Parkinson’s disease (PD)^2–4^. PINK1 possesses an N-terminus mitochondrial targeting sequence constitutively directing the protein from the cytosol to the mitochondria, where it ‘senses’ the structural integrity of the mitochondria. Within a healthy mitochondria, which has an established electronegative membrane potential (Δψ_m_) essential for oxidative phosphorylation, PINK1 is translocated from the outer mitochondrial membrane (OMM) to the inner mitochondrial membrane (IMM). Here it is cleaved by a protease, presenilin-associated rhomboid-like protein (PARL), and subsequently exported back into the cytosol where it is further degraded by the proteasome^5^. However, under conditions of cellular stress where there is loss of Δψ_m_, PINK1 becomes stably expressed on the OMM. Here it phosphorylates specific substrates, most notably ubiquitin and Parkin, leading to ubiquitination of OMM proteins that initiate phagophore formation and clearance of the damaged organelle (or part of) to the lysosome, protecting against cellular stress and apoptosis^6–9^.

Platelets are a highly dynamic blood cell, rapidly responding to structural alterations in the vessel wall to limit blood loss (haemostasis), while exacerbated platelet activity can pathologically lead to vessel occlusion (thrombosis) causing ischemic tissue damage. Once critical feature of platelet function is their procoagulant capacity, where surface-exposed phosphatidylserine (PS) provides a vehicle for localised thrombin generation through interaction with coagulation factors, Va and Xa, forming a fibrin matrix that is essential for haemostasis^10–12^. This procoagulant function is initiated by sustained rises in intraplatelet Ca^2+^ and reactive oxygen species (ROS), which lead to mitochondrial permeability transition pore (mPTP) opening and mitochondrial dysfunction through loss of Δψ_m_^13,14^. Opening of the mPTP is crucial for this procoagulant response as platelets deficient in cyclophilin D (CypD), a critical regulator of the mPTP, have a markedly reduced procoagulant response and loss of Δψ_m_ in response to platelet stimulation^15^. The precise mechanisms linking opening of the mPTP to platelet PS exposure are largely unknown, but it is speculated that heightened Ca^2+^ levels in conjunction with mitochondrial signals derived from mPTP opening (including ROS) act synergistically to regulate this response^14^.

Interestingly, there are a number of studies in PINK1^−/−^ cells (mouse embryonic fibroblasts, mouse cortical neurons and neuroblastoma cells) demonstrating altered mitochondrial dynamics, in particular increased mPTP opening, a reduction in Δψ_m_, altered Ca^2+^ homeostasis and increased sensitivity to oxidative stress due to defects in mitophagy^16–18^. Notably, a recent publication demonstrated an important, protective role for mitophagy in platelets in diabetics^19^. Lee and colleagues identified an upregulation of proteins involved in autophagy (Beclin 1, ATG3/7, LC3I/II) and mitophagy (PINK1, Parkin) in platelets from diabetic patients. The functional relevance of this was further revealed by studies in diabetic mice deficient in PINK1, which displayed enhanced platelet activation and thrombosis compared to wild type (WT) diabetic mice, suggesting PINK1-driven mitophagy alleviates severe cellular stress in platelets. Similarly, it has been recently shown that hypoxia activates mitophagy in platelets, which lowers platelet activity^20^. However, it is not clear to what extent PINK1 alters platelet activity under physiological conditions and whether this may reveal a susceptibility to bleeding or thrombotic complications in otherwise healthy PD patients with loss of function mutations in the *PINK1* gene. We hypothesised that platelets deficient in PINK1 would elicit altered sensitivity to platelet stimulation, in particular their procoagulant response reflected through changes in mitochondrial function. Therefore, the aim of this study was to investigate the role of PINK1 in platelet activation and function using PINK1 knockout (KO) mice.

## Results and Discussion

**Figure 1:**
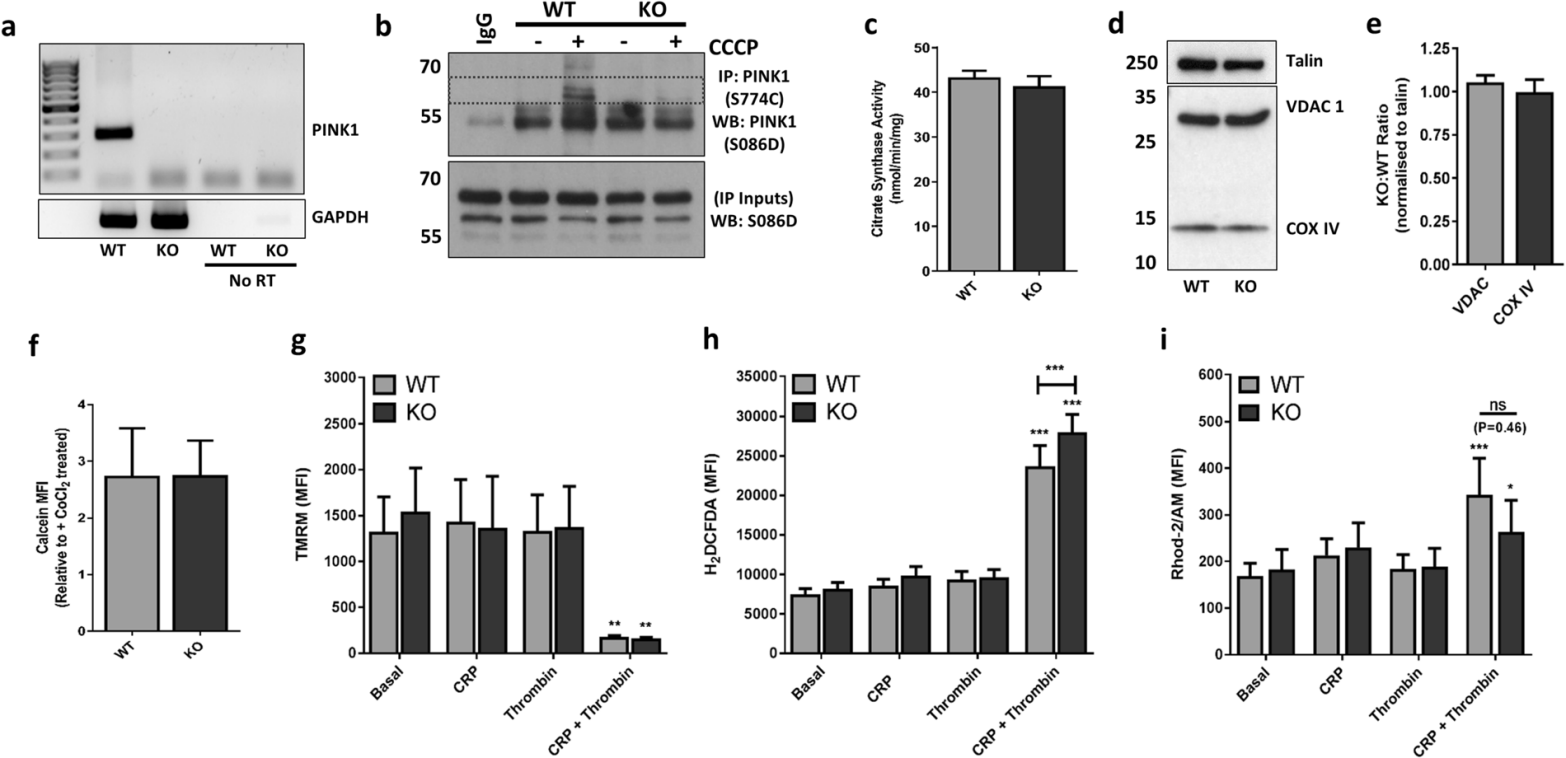
Characterisation of mitochondrial content and function in PINK1^−/−^ platelets. (**a**) Scanned, full-length agarose gel image showing PINK1 mRNA in WT platelets and absence in KO platelets, with cropped GAPDH mRNA gel as loading control. A full-length gel image for GAPDH is provided in Supplementary Fig. S1a. Samples are from the same experiment and the gels were ran in parallel. ‘No RT’ denotes no reverse transcriptase, negative controls during cDNA synthesis step. (**b**) Cropped immunoblots showing PINK1 expression in 10 µM CCCP (6 hours) treated WT, but not KO platelets by IP (upper panel) with loading control of IP inputs by blotting for PINK1 (lower panel). Uncropped blots for PINK1 are provided in Supplementary Fig. S1b. (**c**–**e**) Assessment of mitochondrial mass in platelet lysates by citrate synthase activity measurement and detection of mitochondrial markers, VDAC1 and COX IV, by western blotting, with talin as loading control. Cropped blot images with molecular weight markers are shown, with full-length blot at low and high exposure shown in Supplementary Fig. S1c. Samples are from the same experiment and blot. Densitometric analysis of VDAC and COX IV expression levels was expressed as KO:WT ratio, normalised for loading with talin. (**f**–**i**) Flow cytometry experiments in WT and KO washed platelets (2 × 10^7^/mL) were performed to monitor; (**f**) mPTP opening under basal conditions by calcein quenching with 1 mM cobalt chloride; (**g**) Δψ_m_ with 0.5 µM TMRM; (**h**) reactive oxygen species generation with 5 µM CM-H_2_DCFDA and (**i**) mitochondrial calcium ([Ca^2+^]_M_) levels with 5 µM Rhod-2/AM in response to platelet stimulation for 15 min with CRP (5 µg/mL), thrombin (0.5 U/mL) or combined CRP + thrombin (5 µg/mL + 0.5 U/mL, respectively) in the presence of 1 mM CaCl_2_. Data are mean ± s.e.m (n = 3 for (**c**,**e**), n = 5 for (**f**,**g**, and **i**), n = 7 for (**h**)); *p < 0.05, **p < 0.01, ***p < 0.001 vs. basal (or as indicated); (ns) not significant. Median fluorescent intensity (MFI) values are reported for (**g**–**i**).

Since the identification of PINK1 as a PD-associated gene, there has been a surge in research towards a mechanistic understanding of how this protein functions within eukaryotic cells. Its subsequent discovery as a key regulator of mitochondrial quality control and disposal, coupled to association of mitochondrial dynamics in platelet procoagulant activity, led us to hypothesise that platelets deficient in PINK1 may be susceptible to alterations in function. In the first instance it was important to establish expression of PINK1 in WT platelets. PINK1 mRNA was reliably detected in WT and not KO platelets (Fig. 1a) and corroborates published platelet transcriptome data from Rowley *et al*. who report abundant PINK1 mRNA expression in both human and mouse platelets^21^. We could also confirm protein expression of PINK1, but only by an immunoprecipitation (IP) method in WT platelets treated with the protonophore, carbonyl cyanide 3-chlorophenylhydrazone (CCCP - Fig. 1b – dashed grey box within upper panel). This compound depolarises the mitochondrial membrane, thus stabilising PINK1 from constitutive degradation and is a standard approach for detecting endogenous PINK1^22,23^. Notably, detection of endogenous PINK1 protein in lysates (without IP) from platelets treated with CCCP proved challenging, as bands around the predicted molecular weight of full length (FL)-PINK1 (63 kDa) or cleaved PINK1 fragments (55 and 45 kDa) were either undetectable or present in both WT and KO samples suggesting various commercial PINK1 antibodies cross-react with more abundant proteins in platelets (Supplementary Fig. S2). Consistently, there are numerous reports in the literature citing sensitivity issues associated with detecting endogenous, but not recombinant overexpression, of PINK1 and likely reflects the constitutive degradation of the endogenous protein in healthy cells and antibody sensitivity issues, particularly in tissues with lower expression levels^24–26^.

Haematological analysis of whole blood from PINK1 KO mice did not reveal any significant differences in cell count and or mean platelet volume (MPV) (Table 1). Notably, a study by Kocer *et al*. reported normal platelet count, but an increase in MPV in PD patients^27^. However, disease severity based on Heohn and Yahr scores, was crucial as those patients in the later stage of PD negatively correlated with MPV. It is suggested that variances in inflammatory states throughout PD progression support these MPV changes^27^. However, our analysis of white blood cell counts, as a surrogate marker for inflammation, were not significantly altered in PINK1 KO mice, which is consistent with a previous study reporting no alterations in inflammatory markers in different brain regions of PINK1 KO mice^18^. Subsequent studies set out to assess mitochondrial characteristics and function in PINK1 KO platelets by a variety of standard approaches. Increased mitochondrial mass has been reported in PINK1^−/−^cells^17,18^, but our assessment of citrate synthase activity and expression of the IMM and OMM proteins, COX IV and VDAC, respectively, were unaltered (Fig. 1c–e). Ultrastructural analysis of platelets from PD patients did not reveal any alterations in mitochondria or granules numbers^28^. Furthermore, if there was an increase in mPTP opening in PINK1 KO platelets, as reported in PINK1^−/−^ cells, one would expect an enhancement of the relative calcein fluorescent signal between unquenched (no CoCl_2_) and quenched (+ CoCl_2_) platelet samples, but no significant difference was observed (Fig. 1f). Further experimental assays were performed in platelets stimulated with both collagen-related peptide (CRP, 5 µg/ml) and thrombin (0.5 U/mL), acting via the glycoprotein (GP) VI receptor and protease-activated receptors (PAR, −3/4 in murine platelets), respectively. Dual stimulation with high concentrations of both agonists is necessary to induce loss of Δψ_m_ required for platelet procoagulant activity^15^. Under resting conditions, the Δψ_m_ was comparable between WT and KO platelets as assessed by TMRM staining and platelet activation with dual agonists (CRP + thrombin) induced a similar loss of Δψ_m_ between both genotypes (Fig. 1g). Despite numerous reports of loss of Δψ_m_ in PINK1^−/−^ cells, our result is consistent with our observation that mPTP was unaltered in KO platelets, which is a precipitating event causing loss of Δψ_m_^16^. Interestingly, a subtle, yet statistically significant increase in ROS production, following dual agonist treatment, was observed using the generic detector H_2_DCFDA, a finding which is more in line with observations from PINK1^−/−^ cells (Fig. 1h)^17^. However, experiments using the reported mitochondrial ROS detector, MitoSOX, did not reveal any significant signal enhancement (Supplementary Fig. S3). Finally, mitochondrial Ca^2+^ ([Ca^2+^]_M_) responses were assessed using Rhod-2AM^29^. Significant enhancements of [Ca^2+^]_M_ responses were observed in both genotypes in response to dual agonist stimulation and while there appeared to be a reduced response in KO platelets, it was not statistically significant (Fig. 1i, P = 0.461). It has been reported that agonist-induced rises in [Ca^2+^]_M_ are regulated through the membrane potential-dependent mitochondrial calcium uniporter (MCU), and therefore loss of Δψ_m_ in PINK1^-/-^ cells leads to defects in [Ca^2+^]_M_ uptake^17,30^. However, no changes in Δψ_m_ in PINK1^−/−^ platelets were observed in our experiments and therefore likely explains why the [Ca^2+^]_M_ buffering capacity was not significantly unaltered in PINK1^−/−^ platelets.

**Table 1:** Haematological parameters and platelet surface analysis of glycoprotein receptor levels.

Parameter	WT			KO			P value
	Mean	SEM	N	Mean	SEM	N	
WBC (10^3^/mm^3^)	6.04	0.52	9	5.651	0.55	9	0.617
RBC (10^6^/mm^3^)	11.28	0.58	9	11.06	0.38	9	0.749
Plt (10^3^/mm^3^)	926	61	9	896	36	9	0.686
MPV (µm^3^)	5.51	0.08	9	5.65	0.08	9	0.245
Plateletcrit (%)	0.414	0.038	9	0.409	0.021	9	0.906
GPIIb (MFI)	8507	92	5	8558	77	5	0.678
GPIbα (MFI)	2290	292	5	2203	265	5	0.829
GPVI (MFI)	883	25	5	862	29	5	0.586

Whole blood from WT and KO mice drawn in 4% citrate was analysed on a Horiba Pentra ES60 haematological analyser, with cell counts corrected for dilution in citrate. WBC, white blood cell; RBC, red blood cell; Plt, platelet; MPV, mean platelet volume; PCT, plateletcrit. Washed platelets (2 × 10^7^/mL) from WT and KO platelets were analysed for surface expression of GPIIb, GPIbα and GPVI by flow cytometry. Median fluorescent intensity (MFI) values are reported. Data are mean ± s.e.m and the indicated number (N) of independent experiments. P values were determined by unpaired student’s t-test (two-tailed).

**Figure 2:**
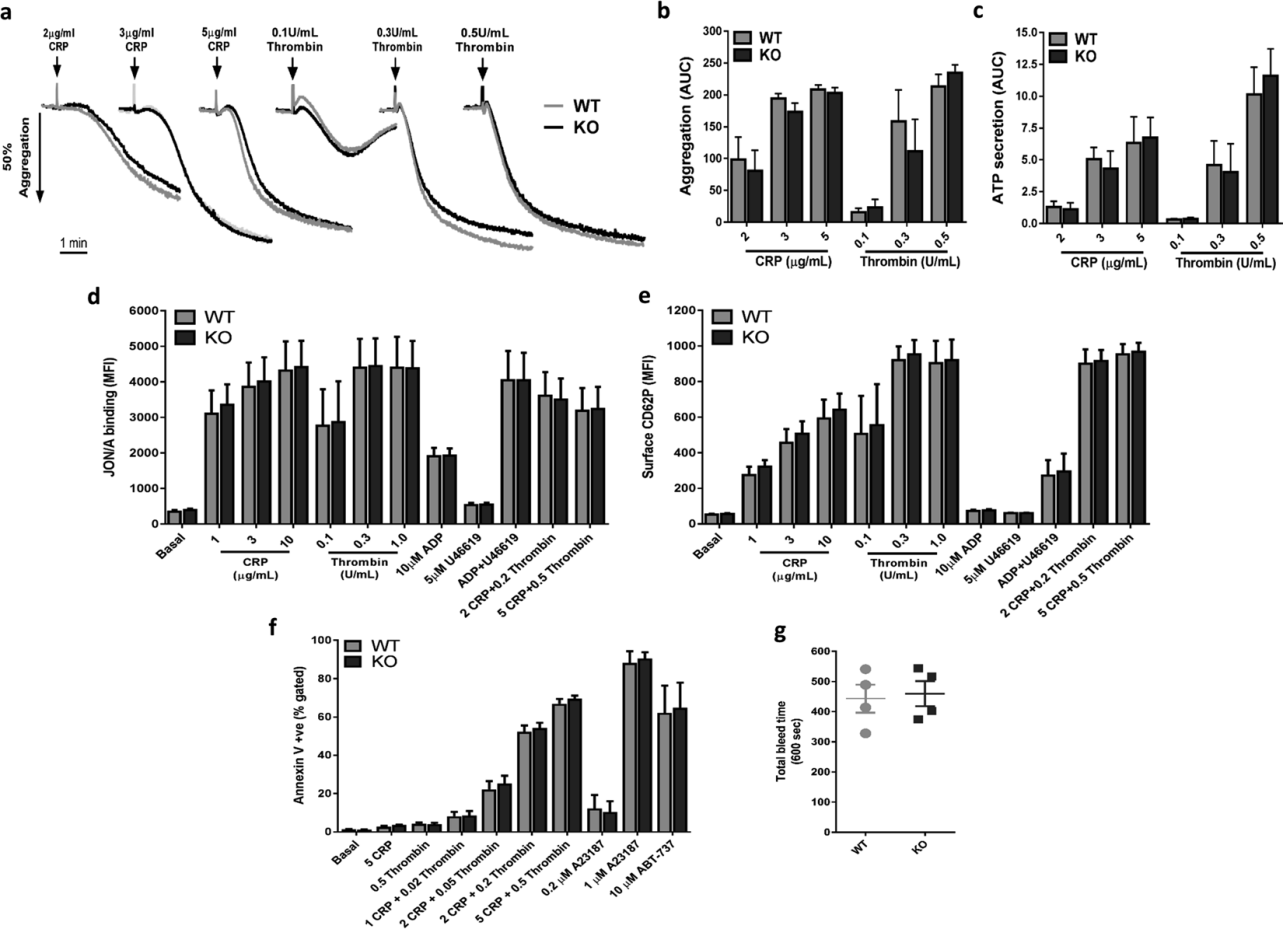
Loss of PINK1 does not alter platelet aggregation, secretion, integrin activation, procoagulant capacity or haemostasis. (**a**–**c**) Washed platelets (2 × 10^8^/mL) from WT and KO mice were assessed by lumi-aggregometry with indicated concentrations of CRP and thrombin. Representative aggregation traces are shown (**a**), with quantified area under the curve analysis of platelet aggregation (**b**) and dense granule secretion of ATP (**c**). (**d**,**e**) Washed platelets (2 × 10^7^/mL) were stimulated with a panel of platelet agonists for 15 min and monitored for integrin α_IIb_β_3_ activation (**d**) and α-granule secretion (**e**) by JON/A antibody binding and P-selectin/CD62P detection antibody, respectively. (**f**) Platelet phosphatidylserine (PS) exposure was monitored in washed platelets (2 × 10^7^/mL) following 15 min stimulation with CRP, thrombin, increasing concentrations of CRP + thrombin (µg/mL + U/mL, respectively), the calcium ionophore, A23187, and the BH3 mimetic ABT-737 (treated for 3 hours), by staining with annexin V-488. Changes in PS exposure are presented as annexin V + ve (positive) events, with basal values set between 1–2% + ve events. (**g**) Tail bleeding times from WT and KO mice were recorded over a 10 min period. Data are mean ± s.e.m (n = 5 for (**a**–**c**), n = 3 for (**d**,**e**), n = 7 for (f-, except ABT-737 treated samples were n = 4), and n = 4 for (**g**)). Median fluorescent intensity (MFI) values are reported for (**d**,**e**).

Despite the lack of clear alterations in platelet mitochondrial indices, subsequent experiments sought to investigate functional responses in PINK1^−/−^ platelets. Importantly, no changes in expression of key platelet plasma membrane receptors in KO platelets were detected (Table 1). Lumi-aggregometry was performed to assess platelet aggregation and dense granule secretion using a range of CRP concentrations (2–5 µg/mL) and thrombin (0.1–0.5 U/mL). However, no significant differences were detected compared to WT platelets (Fig. 2a–c). Further flow cytometry assays assessing platelet integrin α_IIb_β_3_ activation (JON/A binding) and α-granule secretion (CD62P/P-selectin surface exposure) were performed using a panel of platelet agonists at various concentrations, but again no significant differences were observed (Fig. 2d,e). It has been previously shown that loss of cyclophilin D, a key regulator of mPTP opening and platelet procoagulant function, has no impact on platelet aggregation and secretion responses^15^. Therefore the lack of a detectable phenotype in PINK1^−/−^ platelets may not be entirely surprising, but it was initially anticipated that platelet PS exposure (procoagulant function) as assessed by annexin V binding may be altered in KO platelets. However, increasing concentrations of combined CRP (1–5 µg/mL) and thrombin (0.02–0.5 U/mL) did not reveal any defect/enhancement of annexin V binding, while non-receptor-mediated Ca^2+^ entry with the ionophore, A23187, also elicited comparable responses between WT and KO platelets (Fig. 2f). It is also known that an agonist-independent, apoptotic pathway of mitochondrial disruption leading to PS exposure exists in anucleated platelets^31^. Induction of this pathway with the BH3 mimetic, ABT-737, did substantially enhance PS exposure, but the extent of the response was yet again similar in KO platelets (Fig. 2f). Finally, tail vein bleeding times, were also normal in KO mice suggesting that loss of PINK1 does not lead to any haemostatic abnormalities (Fig. 2g).

Overall, our experimental findings on PINK1 KO platelets did not reveal an important physiological role for this protein. The only significant difference detected was a subtle increase in ROS levels following platelet activation, but this did not enhance functional platelet responses tested. This may seem to contradict the paper published by Lee *et al*., who reported heightened thrombotic risk in PINK1 KO mice^19^. However, these mice were diabetic and therefore platelets are already in a ‘primed’ state due to elevated levels of oxidative stress associated with the disease and loss of PINK1 cannot protect platelets (and other cells) through clearance of damaged mitochondria^32^. Pertinent to this finding, it would be interesting to assess if loss of PINK1 in other mouse models of cellular stress, such as bacterial infection or atherosclerosis, support a protective, anti-thrombotic role for mitophagy (via PINK1) in platelets. Notably, these observations raise a number of important questions. First, is PINK1-induced mitophagy only prevalent under stressful conditions *in vivo*? Interestingly, it has been recently demonstrated that basal mitophagy occurs independently of PINK1 in a number of metabolically demanding tissues *in vivo* and is therefore supportive of our observations showing no discernible platelet phenotype under non-diseased conditions^33^. Furthermore, how is basal mitophagy controlled and what relevance does it have to platelet function? Notably, a recent study in platelets identified FUNDC1, a mitophagy receptor protein that localises to the OMM, as an important regulator of basal mitophagy, with platelets from FUNDC1^−/−^ mice having increased mitochondrial mass and decreased mitochondrial function^20^. Platelet aggregation and secretion responses were reduced in FUNDC1^−/−^ platelets suggesting that mitochondrial quality control checks in ‘healthy’ platelets do exist and are functionally relevant to preserve haemostatic properties of platelets. Gautier *et al*. also showed that age is another crucial factor that can influence tissue-specific abnormalities in mitochondrial function due to loss of PINK1 and it therefore possible that studies on aged mice (1–2 years) may uncover basal mitochondrial alterations that influence platelet production and function^18^. It also possible that compensatory pathways initiating basal mitophagy exist in platelets, as previously reported in other cell systems^34^. In concluding, PINK1 appears dispensable for platelet function and mitochondrial regulation in otherwise healthy mice. It is currently unknown to what extent other PD-associated genes involved in mitophagy, such as *Parkin*, *DJ-1*, and *LRRK2* impact on platelet mitochondrial control and function.

## Materials and Methods

### Materials

Thrombin, adenosine diphosphate (ADP), prostaglandin e1 (PGe1) and apyrase grade VII were from Sigma-Aldrich (Poole, UK). Cross-linked collagen related peptide (CRP) was purchased from Professor Richard Farndale (University of Cambridge, UK). Carbonyl cyanide 3-chlorophenylhydrazone (CCCP), Thromboxane mimetic, U46619, and the calcium ionophore, A23187, were from Tocris Bioscience (Bristol, UK). The BH3 mimetic compound, ABT-737 was from Santa Cruz (Insight Biotechnology, Middlesex, UK). For western blotting, the antibodies were as follows: PINK1 (#BC100–494, 1:1000 dilution, NOVUS Biologicals, Abingdon, U.K.), PINK1 (#sc-33796, 1:500 dilution, Santa Cruz), PINK1 (#S774C and #S086D, both 1:500 dilution, Dundee MRC PPU), PINK1 (#6946, 1:1000 dilution), voltage dependent anion channel (VDAC, #4866, 1:1000 dilution) and cytochrome oxidase (COX) IV (#4850, 1:1000 dilution) antibodies were from Cell Signaling Technology (New England Biolabs, Hitchin, UK) and talin antibody (#sc-7534, 1:500 dilution) was from Santa Cruz. Horseradish peroxidase (HRP)-conjugated secondary antibodies were from Jackson Immunoresearch (Stratech Scientific, Glasgow, UK). Fluorescent probes used in flow cytometry assays: tetramethylrhodamine (TMRM, #T668), chloromethyl-2′, 7-dichlorodihydrofluorescein diacetate (CM-H_2_DCFDA, #C6827), MitoSOX™ Red (#M36008) and Alexa Fluor 488-conjugated annexin V (#A13201) were from Molecular Probes (Thermo Fisher Scientific, Loughborough, UK). Rhod-2 acetoxymethyl (AM) ester was from Santa Cruz (#sc-202790) and calcein-AM from Tocris (#5119). Anti-mouse antibodies used in flow cytometry experiments: PE-conjugated JON/A (activated integrin α_IIb_β_3_, #M023-2), FITC-conjugated CD62P (P-selectin, #M130-1), FITC-conjugated Xia.G5 (CD42b, #M040-1) and FITC-conjugated JAQ1 (GPVI, #M011-1) were from Emfret Analytics (Eibelstadt, Germany) and FITC-conjugated MWReg30 (GPIIb/CD41, #GTX76011) was from GeneTex (Source Bioscience, Nottingham, UK). Protease (#11836170001) and phosphatase (#04906845001) inhibitors were from Roche (West Sussex, UK). Unless stated, all other chemicals were purchased from Sigma-Aldrich.

### Mice and washed platelet isolation

A constitutive, *Pink1*^−/−^ (knockout, KO) mouse model on a mixed C57BL/6J-129SvEv^Brd^ background was generously provided by Dr. Miratul Muqit (MRC PPU, Dundee, UK), and were generated as previously described^35^. For breeding, *Pink1*^+/−^ mice were crossed to generate *Pink1*^−/−^ mice and littermate *Pink1*^+/+^ mice, which were used as control (wild-type, WT). All animal studies were approved by the local research ethics committee at the University of Bristol, and mice were bred and maintained in accordance with the UK Home Office regulations and Animals (Scientific Procedures) Act of 1986 (PPL No: 300/3445 held by Prof. Alastair Poole). For experimental procedures, age- and sex-matched mice, 8–20 weeks of age were sacrificed by a gradual rise in CO_2_ inhalation. Blood was drawn from the inferior vena cava into a syringe containing 4% trisodium citrate (1:10 v/v) and washed platelets were prepared as previously described^36^. Unless otherwise stated, 1 mM CaCl_2_ was supplemented immediately prior to platelet stimulation with agonist.

### Aggregation and ATP secretion

Simultaneous monitoring of platelet aggregation and ATP (dense granule) secretion in 240 µl washed platelets (2 × 10^8^/mL) was performed using a Born lumi-aggregometer (Chrono-log® Model 700, Labmedics, Oxfordshire, UK) under constant stirring at 1,000 rpm (37 °C). Luciferin-luciferase reagent (CHRONO-LUME®), to measure released ATP, was added prior to the addition of 5 µl agonist. Post reaction, 1 nmol ATP standard was added to calibrate the reaction and results for both are expressed as area under the curve.

### Platelet mRNA isolation and PCR analysis

Platelet rich plasma (PRP) from 3 x WT or KO mice (~1.2 × 10^9^ platelets) were pooled and passed, via gravity, through leukocyte depletion filters (PALL Corporation, Portsmouth, UK). Filtered PRP was centrifuged at 2,000 rpm in the presence of 1 µM PGe1 and 0.02 U/mL apyrase. The resulting platelet pellet was lysed and platelet mRNA was extracted using the Qiagen miRNeasy Micro Kit (Manchester, UK) as per manufacturer’s instructions. For subsequent cDNA synthesis, 0.1 µg RNA was added as template in the reverse transcription protocol using the Invitrogen Super-Script™ IV VILO™ kit (Thermo Fisher Scientific). For PCR amplification, 2.5 µl cDNA was added to a 25 µl reaction mix containing 0.4 µM forward and reverse primer (final concentration). Thermocycler conditions for PCR were as follows: 5 min at 95 °C, followed by 35 x cycles (0.5 min at 95 °C, 0.5 min at 56 °C, 1 min at 72 °C) and finally 5 min at 72 °C (Bio-Rad, Hertfordshire, UK). Amplified samples were loaded onto 1.5% agarose gels, separated at 100 V for 30 min and visualised using a Syngene G:Box system (Cambridge, UK). PINK1 primers 5′ > 3′ were as follows: forward - TTGCCCCACACCCTAACATC and reverse - CGGACTTGAGATCCCGATGG. GAPDH primers 5′ > 3′ were as follows: forward - ACAAAATGGTGAAGGTCGGTGTGA and reverse – GATGACCCTTTTGGCTCCACCCT.

### Immunoprecipitation (IP) and western blotting

For the IP procedure, 5.6 × 10^8^ platelets/sample, were treated with vehicle (0.1% DMSO) or CCCP (10 µM) for 6 hours at 37 °C, then lysed in 1% Triton X-100 containing lysis buffer (20 mM Tris pH 7.4, 150 mM NaCL, 1 mM EDTA, 1 mM EGTA, supplemented with protease and phosphatase inhibitors). Lysates were clarified at 13,000 rpm (4 °C) for 10 min and 20 µl sample retained for input controls. Lysates were pre-cleared with protein G sepharose (PGS, #6511-1, Generon, Slough, UK) and incubated overnight at 4 °C with 2 µg PINK1 IP antibody (#S774C) or anti-sheep IgG control. Samples were then incubated with PGS for 1 hour at 4 °C, before 3 x wash steps (in lysis buffer) and elution in 1x sodium dodecyl sulfate (SDS) sample buffer. For detection of VDAC, COX IV and PINK1 in lysates, washed platelets (4 × 10^8^/mL) were lysed in 4x SDS sample buffer containing protease and phosphatase inhibitors, snap frozen and stored at −80 °C for further analysis. Lysates (20 µl) were loaded onto 10/12% SDS-polyacrylamide gels and subsequent electrophoresis and immunoblotting procedures were performed as previously described^37^. Densitometry was performed using Image J (Version 1.46, NIH).

### Flow cytometry assays

For measurement of mitochondrial membrane potential (Δψ_m_), reaction oxygen species (ROS) and mitochondrial calcium, washed platelets were dye-loaded with 0.5 µM TMRM, 5 µM CM-H_2_DCFDA and 5 µM Rhod-2 AM, respectively, for 30 min in the dark. For assessment of integrin activation and α-granule release, platelets were mixed with 5 µl PE-JON/A and 2.5 µl FITC-CD62P antibodies, respectively, whereas for phosphatidylserine exposure (PS) analysis, platelets were mixed with 2 µl Alexa Fluor 488-conjugated annexin V. Samples were then stimulated with indicated concentrations of platelet agonists for 15 min at a final platelet concentration of 2 × 10^7^/mL. For assessing mitochondrial permeability transition pore (mPTP) opening in resting platelets, a calcein loading, cobalt chloride (CoCl_2_) quenching technique was used, as CoCl_2_ cannot quench calcein fluorescence within the mitochondrial matrix with an intact mPTP^38^. Platelets (2 × 10^7^/mL) were loaded with 2 µM calcein-AM for 30 min in the dark, before quenching in the absence or presence of 1 mM CoCl_2_ for 10 min in the dark. For measuring platelet surface glycoproteins (GPIIb, GPIbα and GPVI), washed platelets (2 × 10^7^/mL) were incubated for 10 min with 5 µl of their respective antibodies. All samples were gated for 10,000 events on an Accuri™ C6 Plus flow cytometer (BD Biosciences, Oxford, UK) and fluorescent signals detected with the appropriate filters.

### Citrate Synthase Assay

The assay was ran as previously described^39^. In brief, washed platelets (4 × 10^8^/mL) were lysed in 1% triton X-100 buffer containing 150 mM NaCl, 20 mM Tris, 1 mM EDTA, 1 mM EGTA supplemented with protease and phosphatase inhibitors (Roche, UK). Platelet lysate (10 µl) was incubated with 100 mM Tris (pH 8.0), 0.1% triton X-100, 0.1 mM acetyl-coenzyme A and 0.2 mM 5′-dithio-bis-(2-nitrobenzoic acid), before addition of 0.2 mM oxaloacetate to initiate the reaction. This was measured at 37 °C with 10 sec intervals, reading at 412 nm on a Tecan Infinite M200 Pro plate reader (Reading, UK). Enzymatic activity was calculated at 3 min, during steady state and values were corrected for total protein content based on a BCA assay (Thermo Fisher Scientific).

### Tail bleeding assay

Mouse tail bleeding times were performed for 10 min as previously described^40^.

### Statistical analysis

All data were analysed using Graph Pad Prism 7 software. Data are presented as mean ± s.e.m. with the indicated number of independent experiments. Tests for normality included D’Agostino and Pearson or Shapiro-Wilk methods. Statistical differences between samples were determined using two-tailed unpaired t-tests and two-way ANOVA with Bonferroni’s post hoc test. *P < 0.05 was considered statistically significant.

## Electronic supplementary material

Supplementary Figures 1–3

## Data Availability

The data generated and analysed in this study are available from the corresponding author on reasonable request.
