# Supplementary material for: Loss of the mitochondrial kinase PINK1 does not alter platelet function
Source: Sci Rep. 2018 Sep 26;8:14377. doi: 10.1038/s41598-018-32716-4 (PMC6158262; doi:10.1038/s41598-018-32716-4)
Supplement: Supplementary file 1 — Supplementary Figures 1–3 [file 41598_2018_32716_MOESM1_ESM.pdf]

Loss of the mitochondrial kinase PINK1 does not alter platelet function

Tony G. Walsh[1], Marion T.J. van den Bosch[1, 2], Kirsty Lewis[1], Christopher M. Williams[1], & Alastair W. Poole[1]*

From the:

[1] School of Physiology, Pharmacology and Neuroscience, Biomedical Sciences Building, University of Bristol, Bristol, BS8 1TD, U.K.

[2] Current affiliation; InteRNA Technologies BV, Utrecht, 3584 CM, The Netherlands

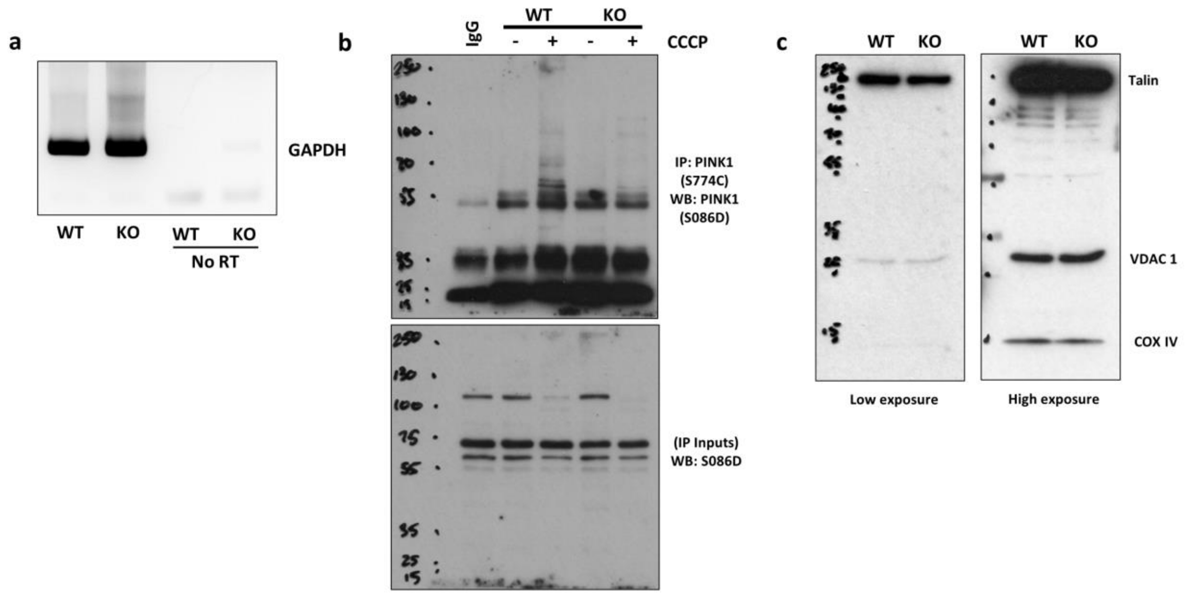

Supplementary Fig. S1. Full length scanned gel and immunoblots. (a) Scanned gel showing detection of GAPDH mRNA loading control in WT and PINK1 KO mRNA. No RT denotes 'no reverse transcriptase' during cDNA step. (b) Scanned full length immunoblots for PINK1 in WT and KO platelets treated with vehicle ('-', 0.1% DMSO) or 10 µM CCCP ('+') for 6 hours at 37°C. Top panel is IP samples, bottom panel is lysate input prior to IP step (c) Scanned full length blot at low and high exposure showing detection of mitochondrial markers: VDAC and COX IV, and talin as loading control in WT and PINK1 KO platelets, with molecular weight markers on the left hand side.

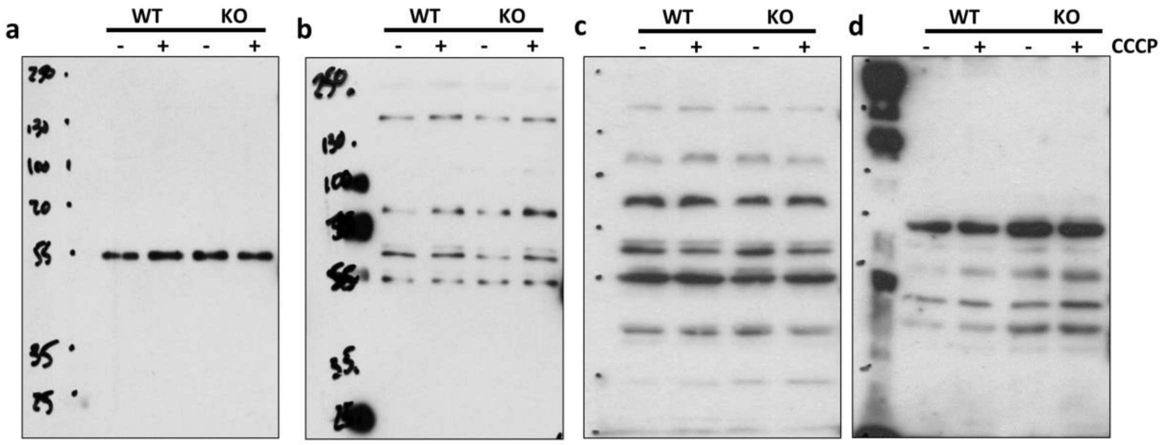

Supplementary Fig. S2. Immunoblotting PINK1 in mouse platelets with 4 different antibodies. Platelets from WT and PINK1 KO mice were treated with vehicle ('-', 0.1% DMSO) or 10 µM CCCP ('+') for 6 hours at 37°C, lysed and immunoblotted for detection of PINK1 using 4 different antibodies: (a) #6946 (Cell Signaling Technology), (b) #BC100-494 (NOVUS Biologicals), (c) #S774C (Dundee MRC-PPU), (d) # sc-33796 (Santa Cruz). Scanned blots show identical band patterns between WT and KO lysates.

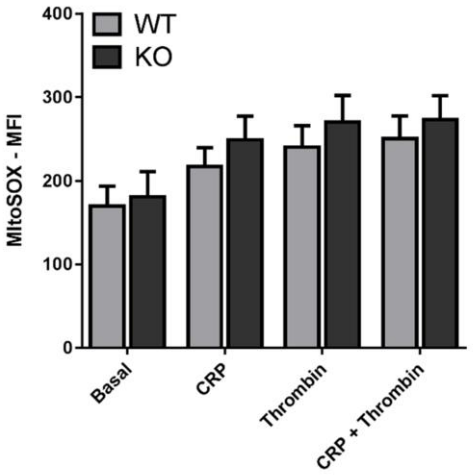

Supplementary Fig. S3. Loss of PINK1 does not alter mitochondrial superoxide generation. Washed platelets (2 x 10^7/mL) from WT and PINK1 KO mice were treated for 15 min with CRP (5 µg/mL), thrombin (0.5 U/mL) or combined CRP + thrombin (5 µg/mL + 0.5 U/mL, respectively) in the presence of 1 mM $CaCl_2$, then dye-loaded for a further 5 min with 5 µM MitoSOX™ Red before flow cytometer analysis. Data are mean ± s.e.m, n=6. Median fluorescence intensity (MFI) values are reported.
